# *Klebsiella ozaenae* Bacteremia in a Kidney Transplant Recipient

**DOI:** 10.1155/2013/493516

**Published:** 2013-04-28

**Authors:** Shree Kumar, Talal Alfaadhel, Meteb M. AlBugami

**Affiliations:** ^1^Department of Medicine, Division of Nephrology, Dalhousie University, Halifax, NS, Canada B3H 1V7; ^2^Queen Elizabeth II HSC-VG Site, Room 5083, Dickson Building, 5820 University Avenue, Halifax, NS, Canada B3H 1V8

## Abstract

Infections remain a dreadful complication after solid organ transplantation. Almost all microorganisms could cause this complication, including unusual ones. We report a 73-year-old patient, with a history of kidney transplant for 38 years on minimum immunosuppression, who presented with high-grade fever and gastrointestinal symptoms. *Klebsiella ozaenae* was isolated from blood cultures. She had a prompt response to antibiotics and recovered completely in a short period. Subsequent evaluation of her nasal cavity and sinuses did not show any abnormalities. *Klebsiella ozaenae* is primarily a colonizer of the oral and nasopharyngeal mucosa, which does not usually cause severe infections. Only 12 cases of *Klebsiella ozaenae* bacteremia have been reported, none of them in the context of solid organ transplant recipient.

## 1. Introduction

Infections are among the commonest causes of morbidity and mortality in solid organ transplant (SOT) recipients and are the second most cause of death in patients dying with functioning allografts [1]. Potential pathogens in this group of patients are diverse, ranging from common pathogens, like community-acquired bacteria and viruses to uncommon opportunistic pathogens that cause infections of clinical significance only in immunocompromised hosts [2]. Infections after SOT may follow a predictable pattern with regard to time elapsed after transplantation [3]. Late infections are often secondary to conventional or opportunistic pathogens; depending on the degree of immunosuppression and environmental exposures.

Dealing with infection in a SOT recipient could be challenging for various reasons. It is more difficult to recognize infection in such patients than it is in persons with normal immune system, as inflammatory responses associated with microbial invasion are impaired by immunosuppressive therapy, which results in diminished symptoms and muted clinical and radiologic findings, and thereby delaying diagnosis. Serologic testing is not generally useful for the diagnosis of acute infection in the SOT recipient since seroconversion is often delayed. Choosing antimicrobial agents is more complex due to the drug toxicities and interactions, which mandate frequent monitoring of immunosuppressive drug levels. Finally, antimicrobial resistance is common in immunocompromised hosts and should be considered in the choice of antimicrobial regimens [2, 3].

*Klebsiella (K.) ozaenae* is a subtype of *K. pneumonia* [4], and has been a known cause of chronic inflammatory disease of the upper respiratory tract [5]. We present the first case of *K. ozaenae* bacteremia in a SOT recipient.

## 2. Case Report

A 73-year-old female, who had a deceased donor kidney transplant 38 years ago, presented to the emergency department with main complaints of fever and gastrointestinal symptoms for 1 day. She reported feeling warm with a measured temperature of 39.6°C at home accompanied by rigors. She felt some nausea and had vomiting and then came seeking medical care. There were no other gastrointestinal complaints, and extensive review of systems failed to identify any potential source of infection. Her other medical history included hypertension, dyslipidemia, stroke, and bioprosthetic aortic valve replacement for aortic stenosis. Her immunosuppressive regimen was azathioprine 75 mg daily and methylprednisolone 2 mg every other day.

Clinical examination reveled a temperature of 39.0°C, pulse rate of 80 beats per minute, blood pressure of 141/70, and oxygen saturation of 97% on room air. Abdominal examination showed normal bowel sound with soft belly and no tenderness. Head and neck, cardiovascular, chest, musculoskeletal, and skin examinations were noncontributory. White cell count was 5400/mm^3^; however, with left shift, hemoglobin was 101 g/L, and creatinine was higher than baseline at 214 umol/L. Urine analysis and chest X-ray were normal. Blood and urine samples were sent for bacterial culture and antibiotic sensitivity.

The patient was admitted to the hospital and started on piperacillin/tazobactam, intravenous fluids and acetaminophen. Within about 18 hours, the blood culture grew *K. ozaenae* sensitive to amoxicillin/clavulanate, cefazolin, ceftazidime, gentamicin, meropenem, trimethoprim/sulfamethoxazole, and tetracycline however resistant to Ampicillin. Urine culture result was negative. 

The patient started to improve within the first 24 hours of hospitalization. She had no more fever, the gastrointestinal symptoms had improved and she was able to tolerate oral food intake. The antibiotic was changed to amoxicillin/clavulanate, and the patient was discharged home in stable condition. Outpatient head CT scan and referral to Ear, Nose, and Throat specialist were arranged due to the nature of this organism. The scan was normal, and the specialist evaluation was unremarkable.

## 3. Discussion

*K. pneumoniae* has three subspecies with homologous DNAs but different biochemical reactions: *K. pneumoniae* subsp. *pneumoniae*, *K. pneumoniae* subsp. *ozaenae*, and *K. pneumoniae* subsp. *rhinoscleromatis* [4]. *K. ozaenae* is a gram-negative, nonmotile, aerobic, encapsulated rod bacterium. It is distinguished from *K. pneumoniae* subsp. *pneumoniae* by a negative reaction to Voges-Proskauer and malonate tests [6]. *K. ozaenae* is an unusual human pathogen and rarely causes serious infections. It has been implicated as the causative agent of ozena: an atrophic rhinitis marked by a thick mucopurulent discharge, mucosal crusting, and fetor [7].

This case represents the first report of *K. ozaenae* bacteremia in a SOT recipient, in the English language literature. The patient had kidney transplantation long time ago and was on minimum immunosuppression. She presented with symptoms of a blood stream infection, however no localizing symptoms or signs. A single pathogen, *K. ozaenae*, was recovered from blood cultures, and after giving a systemic antibiotic, the patient had dramatic improvement. Further evaluation did not show any potential source for this particular pathogen.

*K. ozaenae* bacteremia is a rare clinical problem. In the literature, only twelve cases have been reported [8–16].  Table 1 summarizes the most important features of these cases. Patients were old with an average age of 51.8 years (16–79) and predominantly males. The vast majority of these patients were immunocompromised, a clinical situation that ranged from a medical disease to being on medications that suppress immunity. Chronic rhinitis, which could be the source of the bacteremia, has been reported in some cases. Five out of the eleven patients have died. This high mortality rate could be due to the complicated medical history of these patients, which make them vulnerable to succumb to these conditions even without bacteremia.

Certain risk factors for *K. ozaenae* bacteremia have been identified [11]. These include chronic rhinitis, old age, prior antibiotic usage, immunosuppression, presence of underlying malignancy, and alcoholism. Most of these factors predispose patients for Gram-negative bacterial colonization and/or infection.

*K. ozaenae* has been reported to cause other infections, including meningitis [13], pituitary abscess [17], cholecystitis [15], urinary tract infection [9], and soft tissue infection [9]. From one of the biggest *K. ozaenae* cohort, Goldstein et al. reported a total of 64 isolates of *K. ozaenae* from 36 patients; however, 12 (33%) had documented associated infections (2 bacteremia, 3 urinary tract infection, 1 soft tissue infection, and 6 rhinosinusitis) [9]. Isolates were from upper airways (nasopharynx, throat, and sputum) in 52%, blood in 12%, soft tissues in 11%, urine in 9%, and others 16%.

Antimicrobial susceptibility from previous reports showed that *K. ozaenae* is sensitive to ampicillin in 19% to 26% of the isolates [9, 11]; however, it has a good susceptibility to third generation cephalosporins, fluoroquinolones, and Aminoglycosides. This case showed the same susceptibility. An isolate of extended-spectrum beta-lactamase (ESBL) has been reported on a patient with *K. ozaenae* bacteremia.

In conclusion, SOT recipients are always at risk of blood stream infection even if they have had the organs for long time and even with minimal immunosuppression. All potential pathogens should be considered, including unusual ones, such as *K. ozaenae*.

## Figures and Tables

**Table 1:** Clinical characteristics of *K. ozaenae* bacteremia reported cases.

Reference	Age (y)/sex	Immunocompromising condition	Rhinitis	Outcome
Berger et al. [8]	79/M	No	UNK	Expired
Berger et al. [8]	63/M	ESRD on HD, lung cancer, lupus, prednisone	UNK	Expired
Goldstein et al. [9]	52/M	DM, alcoholic liver disease	Yes	Recovered
Goldstein et al. [9]	19/M	Acute leukemia, chemotherapy	No	Expired
Lewis and Alexander [10]	62/M	No	UNK	Expired
Murray et al. [11]	16/M	Leprosy, prednisone	Yes	Recovered
Murray et al. [11]	74/M	Leprosy	Yes	Expired
Chowdhry and Stein [12]	69/M	DM	UNK	Recovered
Tang and Chen [13]	55/M	Nasopharyngeal carcinoma	No	Recovered
Sarma [14]	16/F	Sickle cell disease	Yes	Recovered
Baig et al. [15]	65/F	DM	No	Recovered
Endailalu et al. [16]	34/M	No	Yes	Recovered

Abbreviations: DM: Diabetes Mellitus; ESRD: End Stage Renal Disease; HD: Hemodialysis; UNK: unkown.
